# Knowledge, practices, and self-reported health outcomes related to chemical use and safety among beauty salons and parlor workers in Polokwane, Limpopo Province

**DOI:** 10.11604/pamj.2024.48.11.39877

**Published:** 2024-05-13

**Authors:** Konyana Edgar Nkoana, Thokozani Patrick Mbonane, Martha Chadyiwa, Renay Helouise Van Wyk, Charlotte Mokoatle, Bheki Magunga, Mpinane Flory Senekane, Phoka Caiphus Rathebe, Shalin Bidassey-Manilal

**Affiliations:** 1 Department of Environmental Health, Faculty of Health Sciences, Doornfontein Campus, University of Johannesburg, Johannesburg, South Africa

**Keywords:** Knowledge, health and safety, chemical hazards, practices, respiratory illnesses, chemical use, chemical safety

## Abstract

**Introduction:**

daily, workers in beauty salons and parlors use different cosmetic products made of various chemicals. This study aimed to determine the knowledge, practices, and self-reported health outcomes related to chemical use and safety among workers in beauty salons and parlors.

**Methods:**

a quantitative, cross-sectional descriptive study was conducted among 145 participants, randomly selected using a simple random sampling design. A self-administered questionnaire was used to assess the Knowledge, practices, and self-reported health outcomes related to chemical safety. Pearson´s correlation was used to assess the correlation between perception, awareness, and other study determinants.

**Results:**

fifty-seven percent of participants had been working with chemicals for more than two years, and only 5% were smokers. There was a positive relationship between age and years of experience in the beauty and salon industry (r= 0.385; p < 0.001), while significant positive correlations between knowledge score with educational levels (r=0.444; p= <0.001) and formal training as a salon and beauty parlor worker (r=0.504; p= <0.001) were also found. Participants also reported symptoms such as headache (43%), nausea (23%), skin irritation (48%), eye irritation (39%), and respiratory illness-related symptoms (62%).

**Conclusion:**

there is a need for health and safety training in beauty salons and parlors, with emphasis on the correct use of personal protective equipment (PPE). The findings of this study may serve as the baseline for the development of safety policies for all beauty salons and parlors in South Africa.

## Introduction

Beauty salons and parlors are establishments where cosmetic professionals use various cosmetic products to offer services like nail care, makeup, body and skincare, style and remove hair, massage, treatments to prevent wrinkles and acne, and hair dying [1]. Due to the popularity and diversity of beauty salons and parlors, their numbers keep expanding at a steady pace. As trends quickly change their needs and demands change too resulting in constantly changing cosmetic products and methods of performing their daily activities, these changes can sometimes be good, and sometimes they can be very bad [2,3]. Products and equipment used in beauty salons and parlors have the potential to emit significantly high concentrations of volatile organic compounds into the surrounding air which the employees are exposed to daily [4]. These products include hair dyes, hair conditioner, and styling lotion which contain benzene and toluene; nail polish and nail glue containing toluene; wig glue containing ethylbenzene; methacrylate is found in artificial nails. Nail polish removers and hairsprays contain xylene; ozone is found in hairsprays and emitted when using steam equipment in anti-ageing and skin treatment; hair dye and nail polish, mascara, blush, eyeshadow, shampoo, and nail treatment contain formaldehyde; carbon monoxide is found during laser hair removal; and nail polish also contains phthalates [5]. All these chemicals are harmful to the environment and humans, as such they have the potential to cause adverse health impacts to exposed individuals, but some pose more severe health impacts than others, like those capable of causing cancer, deformations, and reproductive disorders [6]. The major modes of transmission for all these chemicals are inhalation, absorption, and ingestion [7,8]. Cosmetologists constantly complain that the products they use irritate their skin, eyes, nose, throat, and lungs [9-11].

A study conducted on indoor air quality from four beauty salons in Athens (Greece), showed acute exposure to high ozone concentrations found in salons and mild temporary irritation of the eye and respiratory tract; respiratory tract, and lung inflammation [11,12]. Similar research surveyed cosmetic professionals in Colorado and after working in beauty salons and parlors, 38% of the respondents developed asthma [13]. In another three observational studies done to observe skin conditions (hands) affecting beauty salons and parlors, an excess of 60% of the workers reported they had dermatitis, eczema, and rashes after working in a salon and beauty parlor [14-16]. A study on maternal occupational risk factors for oral cleft conducted in Europe showed that 100 women who worked in beauty salons and parlors and got exposed to cosmetic chemicals in beauty salons and parlors during the first trimester of pregnancy gave birth to babies with reproductive malfunctions (oral cleft) [17]. Occupational health risks and chemical exposures among Asian nail salon workers showed out of the 70% of participants who reported they had been pregnant, 11.7% of them had at least one miscarriage while working in the salon industry [18]. There are about 10500 ingredients in personal care products that are produced and sold globally but only 11% are reviewed and tested for safety [19,20]. There is currently no legal documentation or requirement that summons beauty salons and parlors to disclose the ingredients in their products due to limited tools and resources available to enforce the law [21]. In South Africa, it is a prerequisite for premises to comply with the local authority, in line with relevant By-Laws. All compliant premises are issued with a Health Permit issued by an Environmental Health Practitioner. The National Health Act, 2003 (Act No. 61 of 2003) set National Environmental Health Norms and Standards for Premises and offers guidelines for beauty salons regarding the issuing of health permits [22]. Numerous studies on the knowledge related to chemical use and occupational health and safety have been conducted in other countries in Africa and elsewhere [18,23]. However, there are limited scientific studies in South Africa on the phenomena being studied. The majority of these studies found that workers in beauty salons and parlors have poor knowledge of health and safety issues such as chemical safety [24]. The study aimed to determine the chemical knowledge, practices, and self-reported health outcomes related to the use of cosmetic chemicals among workers in beauty salons and parlors in Polokwane, Limpopo Province. The following objectives were pursued: i) To determine the knowledge of chemical safety among workers in beauty salons and parlors in Polokwane; ii) to determine the practice of chemical use and safety, and factors influencing the practice among beauty salons and parlors in Polokwane; iii) to establish self-reported health outcomes among the study participants.

## Methods

**Figure 1:**
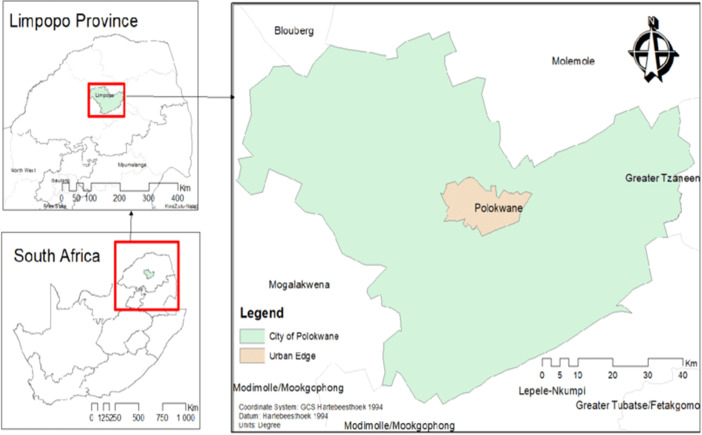
map of Limpopo, with an arrow indicating where the study took place

**Study design and setting:** a descriptive cross-sectional study was conducted among employees in beauty salons and parlors within Polokwane Town. This study was conducted in the central part of Polokwane (Figure 1) which falls within the Limpopo province of South Africa. Polokwane is 106.84 square kilometers with a total population of four hundred and seventy-nine thousand (479000) people 2023. The study was conducted between August and October 2021.

**Study population and sampling:** the study population included employees from the 150 beauty salons and parlors within Polokwane. A statistical software called EPI_INFO version 7.2.4.0 which is designed for the public health community was used to calculate the sample size utilizing 50% expected frequency, an acceptable error margin of 5%, and 1 cluster at 95% confidence level. The determined sample size for the study was one hundred and thirteen (113) salons and beauty parlors. The study included those who were above 18 years old and were working with cosmetic chemicals daily. A Hundred and forty-five (145) participants were selected using a simple random sampling design to participate in the study, allowing every member of the population to have an equal chance to be chosen. A list of participants was received from the salon owners that was added to Microsoft Excel and using the RAND function selected randomly.

**The questionnaire:** data was collected using a questionnaire constructed by the researchers that consisted of close-ended questions. The questionnaire was in English and administered by one researcher (KEN). Literature reviews of previous studies on employees´ health impacts to chemical exposures found in the cosmetic products used in beauty salons and parlors which were similar to the topic of this study were used as a guide to developing a questionnaire that reordered the participants´ daily experiences/effects while working with cosmetic chemical products. The questionnaire collected information about the demographics of the employees, their job history, protective safety measures in salons and beauty parlours, hand hygiene, knowledge of chemical safety and the use of PPE. The questionnaires were administered to all selected participants at their place of employment, and each took approximately 30 to 35 minutes to complete. The questionnaire was structured into sections that answered the identified research objectives.

**Data quality control:** the tool was piloted to ensure its reliability, and validity, and to avoid any bias, it was later edited to refine it and remove all the confusing and repeating or ambiguous questions. The data obtained from piloting the questionnaire was compared with the data from the actual study to determine if the questionnaire recorded the same responses, this ensured that the content of the questionnaire answered all the research objectives and questions.

**Data management and analysis:** data processing included data description, data entry, data cleaning, data editing, and data coding. To ensure the accuracy, completeness, and consistency of the data recorded, all the data was edited and cleaned by checking for duplicated and missing data, renaming, and re-coding relevant variables. Before data analysis, the researcher checked and ensured that all the questionnaires were completed, the incomplete questionnaires were set aside and not included. All 145 participants were included in the data analysis. The data was entered into the IBM SPSS version 28 software where it was cleaned and coded prior analyses. The descriptive data was presented in frequency and percentages using tables and figures. The factors that influence chemical use and safety were determined using the Pearson correlation coefficient, and the p-value was set at 0.005 for statistically significant associations.

**Data assurance:** the participants´ privacy was ensured throughout the study and their confidentiality was kept safe throughout the study due to the nature of the data collection tool (questionnaire) which allowed the participants to complete the questionnaire individually online. Their names were only included on the consent form and no participants´ information was written on the actual questionnaire, this further ensured their anonymity, and their responses were changed into numerals to maintain confidentiality and anonymity because numerals can´t be traced back to the source individual. All the collected data were kept safely in a computer locked with a password and all the online documents including consent forms, information letters, and the actual data were kept on the UJ Drive which is secured with a password known only to the research team. For privacy, no personal information of any participant, and the salons and beauty parlours were published in this study anywhere.

**Ethical considerations:** the study was granted ethical clearance by the University of Johannesburg, Faculty of Health Sciences Research Ethics Committee (REC-1136-2021), while the Capricorn District Municipality Management gave permission to conduct the study in Polokwane. The participants were informed of the study's aim, objectives, their rights (privacy, withdrawal anytime, confidentiality, etc.) and that they did not need to give names and contact details. Thereafter, they signed an informed consent form before participating in the study.

## Results

**Participants characteristics:** a total of 145 full-time beauty salon and parlor workers participated in the study, and there were more females (n=86; 59%) than males (n=59; 41%). Most participants (n=107;74%) were aged between 31 and 40 years old, and most (n=133; 91%) had secondary school as the highest educational level. Fifty-seven percent (n=83) had been working (years of experience) with chemicals for more than two years. Lastly, participants were asked about their smoking status, and only seven (5%) indicated they were smokers. Table 1 provides a detailed description of the sample characteristics. The Pearson correlation showed a positive relationship between age and years of experience (r = 0.385; p < 0.001).

**Table 1:** description of the sample characteristics

Variable	Frequency	Percentage (%)
**Gender**		
Male	59	41%
Female	86	59%
**Age**		
18-30 years	26	18%
31-40 years	107	74%
41-50 years	12	8%
**Educational levels**		
Primary school	1	1%
Secondary school	133	91%
Tertiary education	11	8%
**Years of experience**		
Less than one year	9	6%
1-2 years	53	37%
More than 2	83	57%
**Smoking status**		
Yes	7	5%
No	138	95%

**Figure 2:**
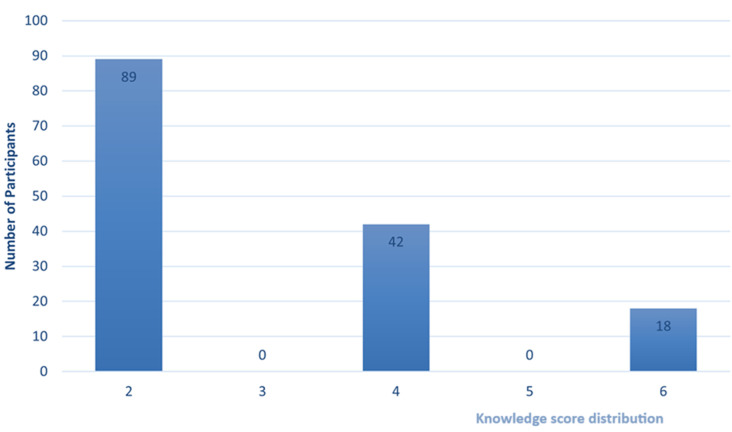
distribution of the knowledge score

**Knowledge of chemical safety:** the participants were asked if they received formal training about their work. There were 15 (10%) participants who had been trained formally. More than half of the participants (n=73; 50.3%) were not trained on the material safety data sheet, and 94% (n=137) did not know how to handle chemicals used in their workplace safely. The participants were not knowledgeable about control measures and actions (n=109; 75%) to follow when in contact with chemicals. Most participants (n=101; 68%) indicated that they would not do anything in case of a chemical burn. Yet, the participants gave the following as examples of control measures: open door (n=88; 61%), Open windows (n=49; 34%), extraction ventilation and air purifier (n=3; 2%), and air dilution system (n=2; 1%). A detailed description of the chemical safety knowledge is in Table 2. In Figure 2 shows the distribution of the knowledge score. The mean of the knowledge score was 2.92, with a standard deviation (SD) of 1.27. While the median was 2.00, and the lowest and highest scores were 2 and 4, respectively

**Table 2:** participants' knowledge of chemical safety

Variable	Frequency	Percentage (%)
**Formal training**		
Yes	15	10%
No	130	90%
**Training on material safety data sheet**		
Yes	72	49.7%%
No	73	50.3%
**Safety on handling chemicals**		
Yes	8	6%
No	137	94%
**Action for chemical burn**		
Nothing	101	70%
Stop using the suspected product	26	18%
Report the incident to the supervisor/relevant authority	14	8%
Make other colleagues aware	4	3%
**Understanding of control measures**		
Yes	36	25%
No	109	75%
**Reported types of control measures**		
Open door	88	61%
Open window	49	34%
Extraction ventilation system	3	2%
Air purifier	3	2%
Air dilution system	2	1%

**Factors influencing knowledge score in the study:** the statistical analysis suggested that two variables strongly influence the knowledge score. There were significant positive correlations between knowledge score with educational levels (r=0.444; p= <0.001) and “having received formal training as a salon or beauty parlor worker” (r=0.504; p= <0.001).

**Figure 3:**
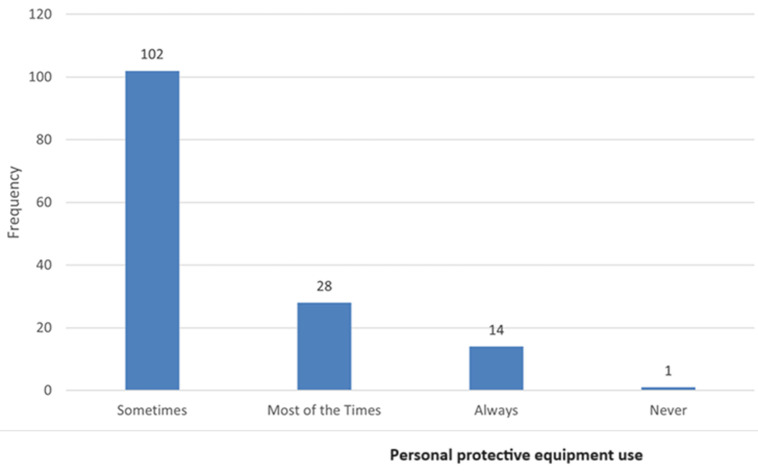
frequency of personal protective equipment use

**Practices related to chemical and personal protective equipment use:** in Table 3 indicates the participants´ practices related to chemicals and PPE use. Most participants indicated that they get their products from registered suppliers (n=144; 99%) and that the products are labelled properly (n=142; 98%). Most participants in the study followed the material safety data sheet (MSDS) instruction (n=130; 89%) and did not handle chemicals with bare hands (n=137; 98%). Most participants (n=141; 97%) wore PPE during working hours. Most participants used all the PPE required, with 90% (n=130) indicating that they use face masks, aprons, and latex protective gloves. Yet others only used either a face mask or latex protective gloves. Figure 3 shows the frequency of PPE use. The use of PPE in the study was as sometimes follows (n=102; 70%), most of the time (n=28; 19%), always (n=14; 10%), and never (n=1; 1%). Furthermore, most participants wash their hands before and after using chemicals (n=143; 98%) or eating (n=140; 94%). Detailed feedback on the practices related to chemical and PPE use is shown in the additional Table.

**Table 3:** participants’ practices related to chemical use and safety

Variable	Frequency	Percentage (%)
**Use of registered suppliers**		
Yes	144	99%
No	1	1%
Cosmetics product labelled		
Yes	3	2%
No	142	98%
**Following material safety data sheet instruction**		
Yes	130	90%
No	15	10%
**Years of experience**		
Less than one year	9	6%
1-2 years	53	37%
More than 2	83	57%
**Hand wash before and after using chemicals**		
Yes	143	99%
No	2	1%
**Hand wash before and after eating**		
Yes	140	97%
No	5	3%
**Access to PPE**		
Yes	141	97%
No	4	3%
**Type off PPE used daily**		
Face masks, aprons, latex protective gloves	130	89%%
Face masks, latex protective gloves	6	4%
Aprons; latex protective gloves	3	2%
Face mask only	2	1.4%
Face masks, aprons	1	0.7%
Latex protective gloves	3	2%
**MSDS: material safety data sheet; PPE: personal protective equipment**		

**Factors impacting the practices related to chemical use and safety:** bivariate correlation analysis showed that using a registered supplier was influenced by the number of years working in a salon or parlor (r=0.206; p= 0.013) and knowledge of the types of control measures that exist (r=0.328; p= <0.001). However, the analysis showed a negative significant correlation between using a register supplier and knowledge score (r= -0.203; p= 0.015). Using cosmetic products that are labelled correctly was linked to buying from a registered supplier (r=0.573; p= <0.001) and knowledge of types of control measures (r=0.204; p= 0.014). Yet, it had a negative link to knowledge of handling cosmetic products bare hand (r= -0.177; p= 0.033) and the need for control measures (r= -0.253; p= 0.009). Washing of hands before and after eating was influenced by using products from a registered supplier (r=0.170; p= 0.041), using correctly labelled cosmetics products (r=0.441; p= <0.001), and washing hands before and after using chemicals. Interestingly, washing hands before and after eating had a negative significant relationship with knowledge score (r= -0.340; p= <0.001).

**Self-reported health outcomes:** participants were asked to indicate if they had experienced some health effects in the last six months. Participants reported experiencing the following symptoms: headache (n=63; 43%), nausea (n=23; 23%), skin irritation (n=75; 48%), eye irritation (n=57; 39%), and respiratory illness-related symptoms (n=90; 62%) such as cough, dry throat, runny nose, and chest pains. Furthermore, participants were asked if they had ever been diagnosed with cancer; no participants reported a cancer diagnosis. While three (2%) reported a family history of cancer. See Table 4 for a detailed descriptive analysis.

**Table 4:** self-reported health effects

Variable	Frequency	Percentage (%)
**Headache**		
Yes	63	43%
No	82	57%
**Nausea**		
Yes	34	23%
No	111	77%
**Skin irritation**		
Yes	70	48%
No	75	52%
**Eye irritation**		
Yes	57	39%
No	88	61%
**Respiratory illness**		
Yes	90	62%
No	55	38%

## Discussion

This study aimed to determine the chemical knowledge, practices, and perceived health effects related to the use of cosmetic chemicals among workers in beauty salons and parlors in Polokwane, Limpopo Province. The results suggest a clear distinction that working in beauty salons and parlors has contributed to employees developing a variety of health impacts. The results show that at least 43% of the participants who have been working with cosmetic chemicals reported experiencing headaches; 23% reported nausea; 48% reported skin irritation; 57% reported eye irritation and 62% reported experiencing respiratory illnesses. This suggests that a significant number of workers in the salon and beauty parlor industry are exposed to potentially hazardous chemicals regularly. This exposure could lead to various health effects, such as respiratory problems [25]. A survey that was done in several nail salons proved that there is a need for appropriate safety measures and control measures to minimize the risks of chemical exposure even in beauty salons and parlors [26]. Only 5% of participants were smokers, smoking can increase the risk of developing certain health problems, such as respiratory illnesses, which may be compounded by exposure to cosmetic chemicals in beauty salons and parlors [27] The lower the percentage of smokers in this population the lower the risk of respiratory illness among salon and beauty parlor workers. However, Pytel *et al*. highlighted that it is essential to note that there may be other factors affecting the health of workers in salon industries, and more research may be needed to determine the precise impact of smoking among people working in beauty salons and parlors [24]. The results suggest that there is a positive relationship between age and years of experience in the beauty and salon industry. Specifically, the correlation coefficient (r) value of 0.385 indicates that there is a moderate positive relationship between these two variables. This was supported by Nguyen *et al*. in their study that proved that as age increases, so does the number of years of experience in salons [28].

This finding is not surprising, as several other studies have shown that individuals who have been in the salon and beauty parlor industry for a longer time would be older/experienced on average [29-31]. The results could have several implications for the salon industry, for example, it could suggest that more experienced workers may have developed better knowledge and skills related to safety and control measures to reduce the risks associated with exposure to chemicals in the workplace [32,3,3]. However, it is important to note that the correlation does not necessarily indicate causation. Other factors could be at play, such as the level of formal training, knowledge, the types of chemicals used and education that the saloon workers have received. There is a significant positive correlation between the knowledge score of the participants and their educational levels and formal training of salon or beauty parlor workers. The correlation coefficient values of 0.444 and 0.504 indicate moderate to strong positive relationships, respectively. This finding implies that the participants who had higher educational levels and more formal training had higher knowledge scores, suggesting that education and training may be critical factors in increasing knowledge about safety and control measures in the beauty and salon industry. Other studies [33,3,4] support this outcome, as the result emphasized the importance of providing adequate training and education to workers in the industry to promote safe working practices and minimize the risks of occupational health hazards associated with exposure to chemicals. Using a registered supplier is influenced by the number of years working in beauty salons and parlors and knowledge of the types of control measures that exist. The correlation coefficient value of 0.206 indicates a weak positive relationship between the number of years working and the use of a registered supplier, while the value of 0.328 indicates a moderate positive relationship between knowledge of control measures and the use of a registered supplier. This finding suggests that workers who have been working in the industry for a long time are more likely to use a registered supplier than those less experienced. Other studies by [35,36] showed that this could be because they have gained more knowledge and experience in the industry and are better aware of the risks associated with using unregistered suppliers. Additionally, beauty salons and parlor workers who have a better understanding of the types of control measures that exist may be more likely to use a registered supplier to ensure that they are purchasing safe and appropriate products [36,37]. The result emphasizes the importance of knowledge and experience in promoting safe working practices and minimizing the risks associated with exposure to chemicals in the beauty and salon industry [37].

**Limitations and strengths:** in this study, the researcher allowed the participants to complete the questionnaires on their own, and this could have invited the response bias. Also, the small sample size restricted the generalization of study findings to the rest of the beauty parlors globally. The study is one of the few studies to correlate factors that influence knowledge and practices on chemical use and safety in a low- and middle-income country. Furthermore, this study accounted for other variables such as smoking which also plays a critical role in the development of respiratory illnesses

The findings from this study can also build new knowledge and fill in the knowledge gap that currently exists on the health and safety of salons and beauty parlor staff especially in low- and middle-income countries like South Africa. Future research could include exploring the effectiveness of targeted educational interventions to enhance chemical safety awareness, investigating long-term health impacts, and assessing the implementation of regulatory measures to promote occupational health and safety within the beauty industry.

## Conclusion

Beauty salon and parlor employees do experience health impacts working with cosmetic products daily, the results highlight this. Thus, according to the Occupational Health And Safety Act and Regulation (85 of 1993), the employer is responsible for ensuring that employees work in an environment that is safe and without risks to the health of the employees, this includes providing training, preventive measures, and PPE to reduce exposures. The study findings indicate a need for ongoing training on chemical use and safety to ensure workers are aware of the potential hazards associated with the chemicals used in their workplace. It also calls for attention from employees, employers and government departments/officials responsible for occupational health and safety to ensure safe chemical practices such as wearing of PPE in the beauty salons and parlor. The contents of this study will likely form the foundation for policy development for salon and beauty parlor workers.

### What is known about this topic

- *It has been documented in various medical reports and research articles that cosmetologists experience negative health impacts from working in beauty salons and parlors;*
- *There isn’t credible legislation and policies that protect cosmetologists from exposure to harmful chemical products they use daily*.

### What this study adds

- *This study will add to the existing knowledge on the health and safety of salon and beauty parlor staff in Polokwane and help educate others on the impacts of the chemicals they use;*
- *This will also shed light on chemical stressors beauty salons and parlor staff get exposed to daily and provides fundamental information for the development of safety policies and guidelines aimed at protecting employees*.
